# *Disc1* Variation Leads to Specific Alterations in Adult Neurogenesis

**DOI:** 10.1371/journal.pone.0108088

**Published:** 2014-10-01

**Authors:** Jayanth S. Chandran, Ilias Kazanis, Steven J. Clapcote, Fumiaki Ogawa, J. Kirsty Millar, David J. Porteous, Charles ffrench-Constant

**Affiliations:** 1 Medical Research Council Centre for Regenerative Medicine, Centre for Multiple Sclerosis Research, University of Edinburgh, Edinburgh, United Kingdom; 2 Neurosciences, University of Cambridge, Department of Veterinary Medicine, Cambridge, United Kingdom; 3 Institute of Membrane and Systems Biology, University of Leeds, Leeds, United Kingdom; 4 Medical Genetics Section, Molecular Medicine Centre, Institute of Genetics and Molecular Medicine, University of Edinburgh, Edinburgh, United Kingdom; Louisiana State University Health Sciences Center, United States of America

## Abstract

*Disrupted in schizophrenia 1* (DISC1) is a risk factor for a spectrum of neuropsychiatric illnesses including schizophrenia, bipolar disorder, and major depressive disorder. Here we use two missense *Disc1* mouse mutants, described previously with distinct behavioural phenotypes, to demonstrate that Disc1 variation exerts differing effects on the formation of newly generated neurons in the adult hippocampus. *Disc1* mice carrying a homozygous Q31L mutation, and displaying depressive-like phenotypes, have fewer proliferating cells while *Disc1* mice with a homozygous L100P mutation that induces schizophrenia-like phenotypes, show changes in the generation, placement and maturation of newly generated neurons in the hippocampal dentate gyrus. Our results demonstrate *Disc1* allele specific effects in the adult hippocampus, and suggest that the divergence in behavioural phenotypes may in part stem from changes in specific cell populations in the brain.

## Introduction

*DISC1* was originally identified as a gene disrupted by a balanced chromosomal translocation t(1;11)(q42.1;q14.3) in a large Scottish family with several major mental disorders including schizophrenia, bipolar disorder and recurrent major depressive disorder [1]. DISC1 is an intracellular scaffold protein that binds to a number of proteins contributing to different signaling pathways [2]. These include interactions with GSK3β and phosphodiesterase 4 (PDE4) at the N-terminus, and NDEL1 and LIS1 at the C-terminus. [3]–[5]. Each of these interacting proteins have been implicated in neural development; GSK3β regulation by wnt signaling enhances neural progenitor proliferation [6], mutations in NDEL1 and LIS1 cause neuronal migration defects [7], [8], and the DISC1-PDE4 complex can modulate the NDEL1/LIS1 interactions [3]. Overexpression of several human DISC1 variants associated with neuropsychiatric phenotypes in cell lines *in vitro* or in embryonic cortical progenitors *in vivo* fails to stimulate cell proliferation and activate β-catenin activity to the same extent as overexpressing wild-type DISC1 [9]. Knockdown of DISC1 in mice leads to reduced cell proliferation and neuronal migration defects in the hippocampus due to perturbations in GSK3β and Ndel1 respectively [4], [10]. Collectively, these results suggest a hypothesis that alterations in DISC1 structure, either due to distinct mutations or individual variations, differentially affect downstream pathways, which might contribute to the different facets of psychiatric disease seen in patients with abnormalities in *DISC1*.

We previously described two independently derived ENU-induced mouse *Disc1* missense mutants which provides an opportunity to test this hypothesis, since they exhibit distinct behavioral abnormalities related to depression (*Disc1* ^31L/31L^) and schizophrenia (*Disc1* ^100P/100P^), and affect binding of DISC1 to PDE4 and GSK3β [11], [12]. In the embryonic cortex, both *Disc1* mutations lead to reductions of neural progenitors, and to mispositioning of neurons in the cortical layers, but the effects of the mutations in adult mice, and in the hippocampus remain unknown [13]. Here, we investigate the effects of the two germline *Disc1* mutations in the hippocampus of adult mice, and determine whether the mutations differentially affect cell proliferation and neuronal migration. Our data suggest that the homozygous Q31L mutation reduces cell proliferation and the homozygous L100P mutation induces deficits in the generation, positioning, and maturation of new neurons in the hippocampus.

## Results

### DISC1 expression not altered by missense mutations

**Figure 1:**
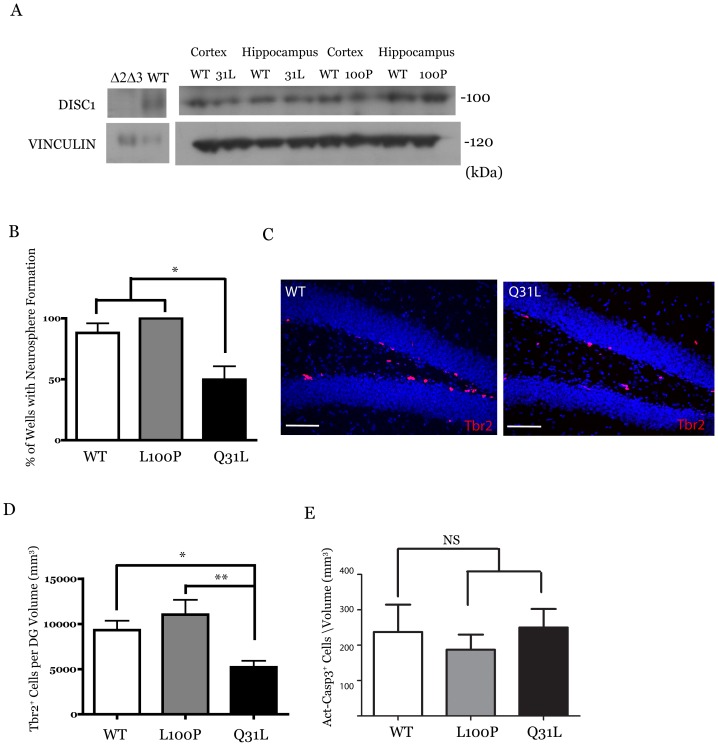
Deficits in cell proliferation are restricted to *Disc1* ^31L/31L^ mice. (**A**) Missense mutations do not affect expression of full length DISC1 in tissue lysates taken from the cortex and hippocampus, as shown using a C-terminal DISC1 antibody that recognizes a specific 100 kDa band in the adult mouse tissue brain homogenates that are absent in *Disc1* ^Δ2Δ3^ mice. (**B**) Significantly fewer primary neurospheres (P = 0.007; *post-hoc* Bonferroni p<0.05) derived from dissociated adult hippocampal cells in *Disc1* ^31L/31L^ mice (n = 6) compared with either wild-type (n = 6) or *Disc*1^100P/100P^ (n = 4) mutants. (**C**) Confocal z-stacks of mouse sections labeled with an antibody raised against the neural progenitor marker Tbr2 (red) and nuclei label Hoechst 33242 (blue) indicate that *Disc1* ^31L/31L^ mutants (n = 8) have significantly (**D**) fewer Tbr2 labelled cells (ANOVA, P = 0.014) than either wild-type (n = 9, *post-hoc* Bonferroni p<0.05) or *Disc1* ^100P/100P^ (n = 9, *post-hoc* Bonferroni p<0.05) mice. (**E**) Cell death as measured by activated caspase-3 immunoreactivity was not significantly different between genotypes (ANOVA, p = 0.74, n = 4 wild type, *Disc1* ^100P/100P^; n = 5 *Disc1* ^31L/31L^; Scale bar 100 µm. Data presented as mean ± SEM.

Due to the report that many commercially available DISC1 antibodies fail to accurately detect the major DISC1 isoform [14], [15], we used an in-house polyclonal C-terminal antibody (see Material and methods) to detect DISC1 in the cortex and hippocampus from the DISC1 mutant and control mice. We verified the specificity of the antibody by showing a unique band close to 100 kDa that was absent in mice with a targeted disruption in exons 2 and 3 (*Disc1* ^Δ2Δ3^) [14] (Figure 1A). When we used this antibody on brain lysates from the DISC1 mice, we found no differences in DISC1 expression between the *Disc1* ^31L/31L^ or *Disc1* ^100P/100P^ mice compared to age-matched wildtype controls (Figure 1A).

### Loss of neural progenitors in *Disc1* ^31L/31L^ mice

Given that several disease associated *DISC1* variants down-regulate wnt signaling and disrupt neural progenitor proliferation [9], we examined whether either *Disc1* missense mutation could affect proliferation in the adult mouse hippocampal dentate gyrus. We initially looked at the efficiency of dissociated adult hippocampal cells in generating primary neurospheres *in vitro* with a colony forming assay to assess whether any proliferation deficits were present (Fig. 1B). Within the adult hippocampus, the majority of neurospheres are formed from cells expressing Hes5, which is expressed in neural stem cells and subsequently down regulated in more neuronally committed progenitors [16]–[18]. Notably, Hes5 deficient cells are unable to form adult primary neurospheres, and so neurosphere formation in the adult hippocampus is an index of the proliferative capacity of progenitors that have not committed to a neuronal fate [17]. When we cultured adult hippocampal cells from the *Disc1* mutant mice at a density of 1000 cells/µl in a 96-well plate format, we noted a significant deficit in *Disc1* ^31L/31L^ mice compared to both wild-type and *Disc1* ^100P/100P^ mice in the percentage of wells with neurospheres formed (WT (n = 6) 88.0%±8.0, *Disc1* ^100P/100P^ (n = 4):98.8%±1.3, *Disc1* ^31L/31L^ (n = 6): 49.7%±11.0, mean ± SEM; ANOVA, F(2,18) = 6.628, P = 0.007; *post-hoc* Bonferroni p<0.05).

Within the adult SGZ, multipotent neural stem cells generate new neurons through the generation of more fate restricted progenitors. To assess whether the cell proliferation deficits induced by the homozygous Q31L mutation extended to neuronally committed progenitors, we labeled cells in the SGZ with an antibody against T-box brain gene 2 (Tbr2), which is expressed by the majority of dividing progenitors that differentiate into neurons in the SGZ, and are therefore a marker for the intermediate progenitors cells (IPCs) that arise from NSC divisions [19]. We noted a significant deficit in Tbr2^+^ cells (Fig. 1C–D) only in *Disc1* ^31L/31L^ mice (WT (n = 9): 9158±979.9, *Disc1* ^100P/100P^ (n = 9):10512±1611, *Disc1* ^31L/31L^ (n = 8): 5213±650.2, Tbr2^+^ cells/mm^3^ dentate gyrus; ANOVA F(2,23) = 5.207, P = 0.014) compared to both wild-type controls (*post-hoc* Bonferroni p<0.05) and *Disc1* ^100P/100P^ mutant mice (*post-hoc* Bonferroni p<0.05), demonstrating a general proliferation defect caused by the Q31L mutation. While the vast majority of Tbr2 immunopositive cells were restricted to the SGZ, we detected a few Tbr2^+^ cells in the hilus and dentate gyrus molecular layer irrespective of *Disc1* genotype, which was detected in hippocampal sections in reports from other groups [20], [21]. This suggests that there are either a few undifferentiated progenitors normally residing in the granule cell layer, or that some postmitotic neurons retain Tbr2 activity. To determine whether the loss of neural progenitors in *Disc1* ^31L/31L^ mice reflects an enhanced cell death, we labeled tissue sections with an antibody detecting activated caspase-3 to label apoptotic cells in the dentate gyrus. We, however, found no significant change in cellular apoptosis (Fig. 1E, ANOVA, p = 0.74, n = 4 wild type, *Disc1* ^100P/100P^; n = 5 *Disc1* ^31L/31L^) between the *Disc1* mutants and wild-type controls, suggesting that the loss of Tbr2^+^ cells in the *Disc1* ^31L/31L^ mice was more likely associated with cell proliferation defects.

### *Disc1* ^100P/100P^ mutants have fewer immature neurons in the SGZ

We next investigated whether the loss of neural progenitors in the SGZ of the *Disc1* ^31L/31L^ mice would lead to changes in the neurons that they differentiate into. The specific role of DISC1 in newly generated neurons in the SGZ is unclear, since neurons display evidence of excessive dendritic branching in mice with an acute DISC1 knockdown, while neurons have limited arbors in mice expressing a truncated DISC1 protein [15], [22].

**Figure 2:**
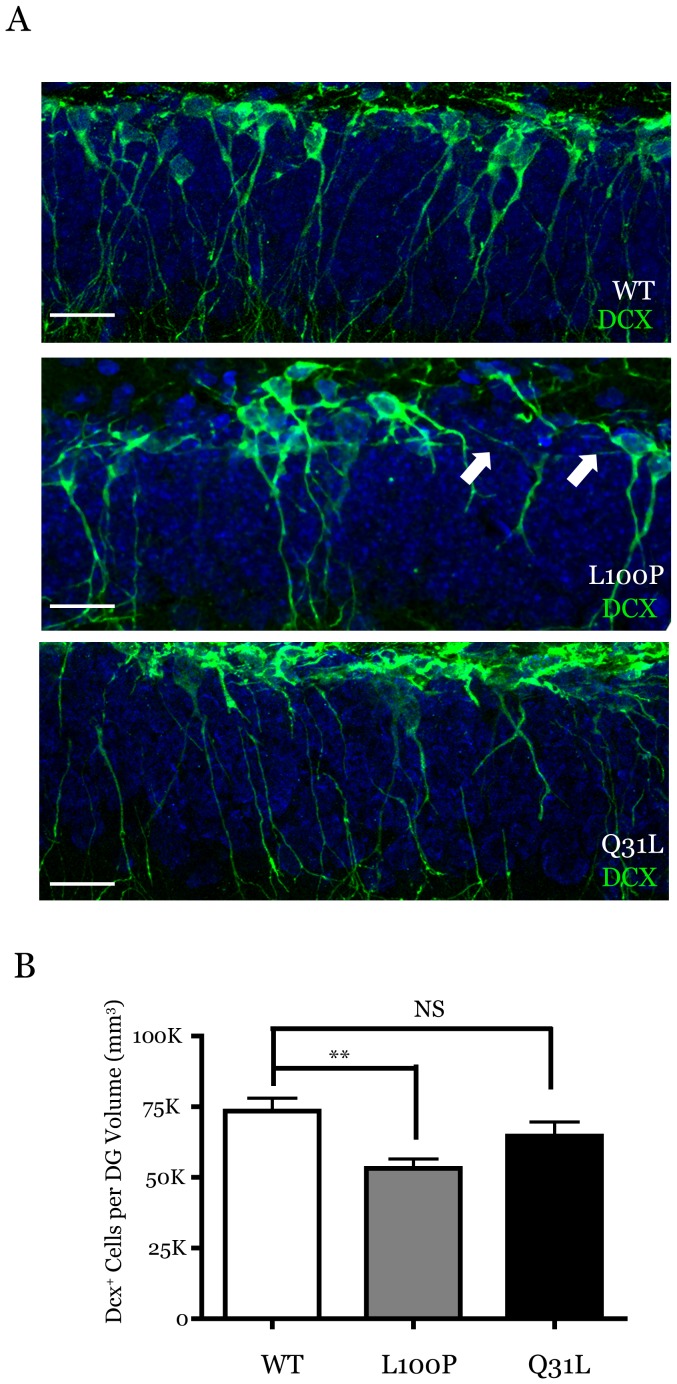
Loss of immature neurons in *Disc1* ^100P/100P^ mice. (**A**) Immunolabelling of immature neurons residing in the subgranular zone with an antibody raised against doublecortin (DCX) identify frequent gaps (arrows) in DCX staining solely in *Disc1* ^100P/100P^ mice that reflect a significant loss (**B**) of DCX^+^ cell bodies compared to wild-type controls (*post-hoc* Bonferroni p<0.05; n = 8 wild type, n = 5 *Disc1* ^31L/31L^. n = 6 *Disc1* ^100P/100P^). Data presented as mean ± SEM. Scale Bar (A) 100 µm (B) 25 µm

To recognize immature neurons in the SGZ, we labeled cells with an antibody to doublecortin (DCX), which is expressed in postmitotic immature neurons [23]. When we examined the morphology of the cells labelled by DCX, we observed numerous gaps in the SGZ layer in *Disc1* ^100P/100P^ mice compared to wild-type controls and *Disc1* ^31L/31L^ mice, resulting in fewer neuronal processes extending across the granule cell layer (Figure 2A). This suggested that the number of DCX^+^ cells were reduced in the *Disc1* ^100P/100P^ mice. To confirm this, we quantified the number of DCX^+^ cell bodies in the SGZ for the *Disc1* mice (Fig 2B, WT (n = 8): 73474±4590, *Disc1* ^100P/100P^ (n = 6): 53209±3364, *Disc1* ^31L/31L^ (n = 5): 64603±5025 DCX^+^ cells/mm^3^ dentate gyrus, mean ± SEM), revealing a significant (ANOVA F(2,67) = 5.374, p = 0.0085; *post-hoc* Bonferroni p<0.05; n = 8 WT, n = 5 *Disc1* ^31L/31L^. n = 6 *Disc1* ^100P/100P^) difference between only the *Disc1* ^100P/100P^ mutant mice and wild-type controls. Although the total number of DCX^+^ cells were reduced in number, the effect of the *Disc1* ^31L/31L^ mutation did not reach statistical significance.

As DCX^+^ neurons functionally integrate and mature into the granule cell layer of the dentate gyrus, we next investigated if a correlation existed between the loss of DCX^+^ cells and a deficit in mature neurons as measured by dentate gyrus thickness. A one-way ANOVA revealed significant thinning (13.9% loss) of the dentate gyrus only in *Disc1* ^100P/100P^ mice (Table 1), though a Student's t-test performed between *Disc1* ^31L/31L^ mice and wild-type controls suggested a trend (p = 0.056) towards thinning (8.7%). Importantly, differences in hippocampal morphology for the *Disc1* ^100P/100P^ mice were specific to the dentate gyrus, as we observed no significant changes (ANOVA, p>0.3) in CA1 or CA3 thickness or hippocampal height or width for either *Disc1* mutant (Table 1). These data support a strong effect of the *Disc1* L100P mutation on neurons in the dentate gyrus.

**Table 1:** Hippocampal Neuroanatomical Measurements between *Disc1* ^31L/31L^, *Disc1* ^100P/100P^ and wild-type mice.

Measurement	Wild-type (n = 9)	Disc1^31L/31L^ (n = 7)	Disc1^100P/100P^ (n = 7)
Dentate thickness (µm)	99.7±2.6	91.0±3.4	85.8±4.9
CA1 thickness (µm)	64.4±2.1	63.0±2.6	68.3±1.4
CA3 thickness (µm)	114.1± 6.6	111.3±2.6	108.3±8.2
Hippocampal width (mm)	2.26±0.12	2.51±0.07	2.23±0.18
Hippocampal height (mm)	1.18±6.6	1.22±0.03	1.24±0.03

A significant difference only in dentate granule cell layer thickness between wild-type and *Disc1* ^100P/100P^ mice was noted (ANOVA F(2,20) = 4.07, P = 0.032; *post-hoc* Bonferroni p<0.05), though Student's *t* test indicated a strong correlation (p = 0.06) between wild-type and *Disc1* ^31L/31L^ mice. Within the hippocampus, the thinning of the granule cell layer in the mutants is specific to the dentate gyrus, as no significant differences are observed between genotypes in the thickness of the pyramidal cell layer in the CA1 and CA3 regions, and the overall height and width of the hippocampus. Data presented as mean ± SEM.

### Ectopic migration of a select population of DCX^+^ cells in *Disc1* ^100P/100P^ mice

**Figure 3:**
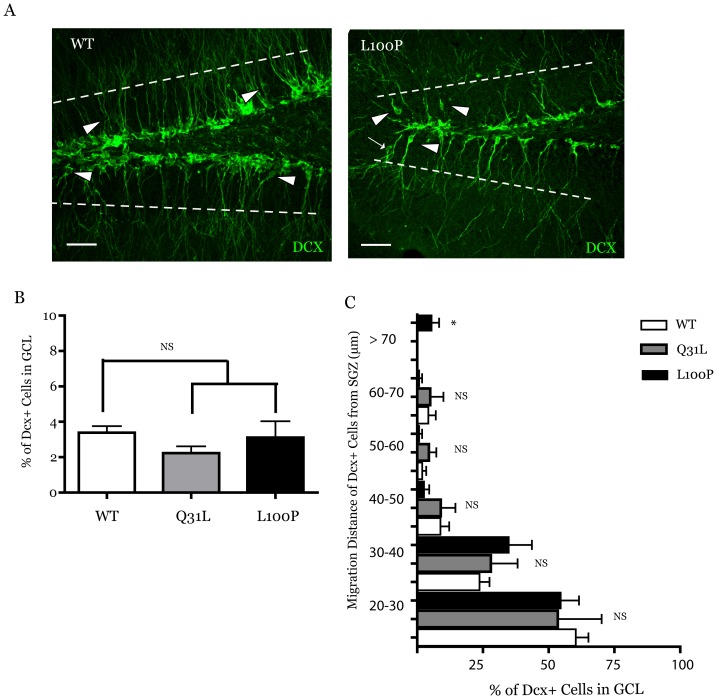
Ectopic migration of select populations of immature neurons in *Disc1* ^100P/100P^ mice. (**A–C**) An analysis of doublecortin (DCX) positive cells prematurely migrating away from the subgranular zone (SGZ) (**A**, upper boundary of GCL demarcated with dashed line) reveal no significant (**B**) differences (ANOVA, p = 0.35, n = 7 wild type, n = 5 *Disc1* ^31L/31L^, n = 5 *Disc1* ^100P/100P^) between genotypes. Arrowheads point to DCX^+^ cell somas positioned in the GCL within 70 µm of the SGZ, while the arrow in *Disc1* ^100P/100P^ section shows DCX^+^ cell positioned greater than 70 µm away from SGZ. (**C**) Binning of DCX^+^ cells in the GCL into 10 µm distance segments from 20 µm away from the SGZ reveal a small but significant (ANOVA F(2,14) = 5.180, p = 0.021; post-hoc Tukey p<0.05; n = 7 wild type, n = 5 *Disc1* ^31L/31L^, n = 5 *Disc1* ^100P/100P^) percentage of cells that migrate a distance greater than 70 µm in the *Disc1* ^100P/100P^ mice compared to wild-type and *Disc1* ^31L/31L^ mice. Data presented as mean ± SEM. Scale Bar 50 µm

Mice expressing a truncated N-terminal DISC1 fragment have a loss of DCX^+^ cells, altered positioning of immature neurons in the granule cell layer, and a reduction in the dendritic complexity of the DCX^+^ immature neurons, suggesting that alterations in DISC1 can affect multiple aspects of neuron morphology [15]. As we had identified changes in the number of the DCX^+^ in *Disc1* ^100P/100P^ mice, we next analyzed the positioning of immature neurons in the SGZ (Fig. 3A, dashed lines indicate upper granule cell layer boundary). While the majority of DCX^+^ cell somas resided neatly along the SGZ, a small percentage of DCX^+^ somas were positioned away from the cells (Fig. 3A, arrowheads) in all the *Disc1* genotypes (Fig. 3B, WT (n = 7): 3.39%±0.36, *Disc1* ^100P/100P^ (n = 5): 3.11%±0.92, *Disc1* ^31L/31L^ (n = 5): 2.23%±0.39, mean ± SEM). When we examined the positioning of these migrating immature neurons by binning the distances from the SGZ into 10 µm sections, we noted that a small percentage of these DCX^+^ cells in the *Disc1* ^100P/100P^ (5.63%±2.75, mean ± SEM) migrated further than 70 µm away from the majority of DCX^+^ somas in the SGZ (Fig. 3A, arrow), which was significantly further than either wild-type or *Disc1* ^31L/31L^ mutant mice (Fig. 3C, ANOVA F(2,14) = 5.180, p = 0.021; post-hoc Tukey p<0.05; WT (n = 7), *Disc1* ^100P/100P^ (n = 5): *Disc1* ^31L/31L^ (n = 5)). These results indicate that in *Disc1* ^100P/100P^ mice, a select population of immature neurons ectopically position away from the SGZ.

### Immature neurons in *Disc1* ^100P/100P^ mutants have less complex dendritic arbors

**Figure 4:**
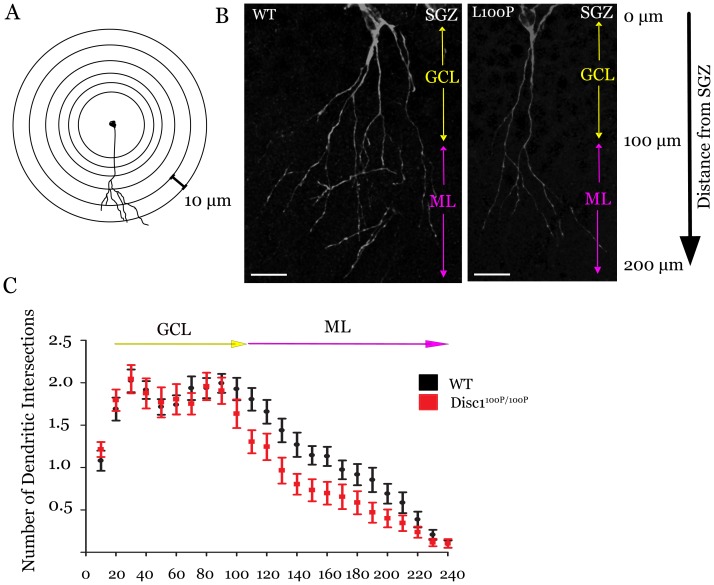
Sholl analysis of immature neurons indicates decreased complexity of the dendritic arbors in *Disc1* ^100P/100P^ mice (A–C). The complexity of dendritic arbors from the *Disc1* cohort was assessed using Sholl analysis to determine the number of dendritic intersections crossing equidistant concentric circles (**A**) extending from the cell somas of neurons labeled with doublecortin. (**B**) High magnification (40x) z-image projections of DCX^+^ neurons with primary processes extending across the granule cell layer (GCL) and into the molecular layer (ML) illustrate the loss of complexity in *Disc1* ^100P/100P^ mice that are confirmed through linear Sholl analysis (**C**) (Two-way RM-ANOVA, p = 0.0003; n = 5 animals each genotype) of dendrites from immature neurons extending from the subgranular zone (SGZ). Scale bar 25 µm. Data presented as mean ± SEM.

Finally, we investigated the dendritic complexity of immature neurons in the *Disc1* cohort using Sholl analysis [24] to count the number of dendritic intersections crossing a series of concentric circles of increasing size centered at the DCX^+^ cell soma (Fig. 4A). We specifically focused on DCX^+^ neurons that extended primary axons into the granule cell layer (and perpendicular to the SGZ), and generated at least one dendritic arbor to exclude the population of neurons that either extended smaller axons parallel to the SGZ or had not matured and generated dendritic morphology. In the wildtype and *Disc1* ^31L/31L^ mice, DCX^+^ neurons displayed highly branched dendritic arbors extending across the granule cell layer into the molecular layer which contrasted with those in the dentate gyrus of *Disc1* ^100P/100P^ mutant mice, which exhibited fewer branches, especially within the molecular layer (Fig. 4B). When we quantified the number of dendritic intersections, we observed a significant reduction in dendritic complexity in Disc1^100P/100P^ mice, but not Disc1^31L/31L^ mice (Fig. 4C, Two-way repeated measures ANOVA, genotype x distance interaction F(25,950) = 2.320, p = 0.0003; n = 5 animals each genotype with between 35–75 neurons measured per animal). We conclude that within the hippocampal dentate gyrus, the Disc1 Q31L mutation affects all the proliferating progenitors, while the L100P mutation selectively affects the postmitotic neurons.

## Discussion

*Disc1* is an attractive gene to study the relationship between disease and cellular phenotypes in the hippocampus due to evidence for *DISC1* as a genetic risk factor for a spectrum of major mental disorders, and because mouse *Disc1* repression has been shown to affect neural progenitor proliferation and neuron specification in the dentate gyrus [4], [22]. We have delineated mutation specific roles for mouse DISC1 in regulating both cell proliferation in the SGZ, and the subsequent migration and maturation of immature neurons in the SGZ. The evidence points to the Q31L and L100P mutations in *Disc1* affecting different cell populations in the SGZ, most likely through selectively aberrant cell signaling. Our results support evidence that cognitive and structural changes in the brain are associated with *DISC1* variation [25].

Previously, acute *Disc1* knockdown in the adult mouse dentate gyrus induced a loss of proliferating cells and generated both schizophrenia-related and depression-related phenotypes [4]. Interestingly, in these mice, Mao et al (2009) also noted aberrant neuronal positioning into the granule cell layer and enhanced dendritic complexity of granule cell neurons that supported an earlier study focused on the maturation of newly generated neurons following *Disc1* knockdown [22]. Our results, however, support data suggesting that marked changes in the early progenitor proliferation exert a minimal effect on the SGZ immature neuron population, as the DCX labeled neurons in the *Disc1* ^31L/31L^ mice appear to be similar to wild-type controls despite the loss of proliferating progenitors in the same mice [26]. Additionally, the *Disc1* ^100P/100P^ mice, in contrast to the mice described following shRNA-mediated *Disc1* knockdown, have a loss of dendritic branching and neuronal maturity that are consistent with mice expressing a truncated DISC1 protein due to germline disruption of *Disc1* affecting exons 7 and 8 [15], [27]. Our findings likely differ from the shRNA-mediated approach due to the timing of the *Disc1* disruption, as our colony of *Disc1* mutant mice carry the missense mutations through the germline. Supporting this, early postnatal, but not adult, induction of a C-terminal human DISC1 protein in mice leads to a loss of dendritic complexity of neurons in the adult hippocampus [28].

Interestingly, mice lacking *Npas3*, a genetic risk factor for schizophrenia, have SGZ proliferation deficits that accompany a thinning of the dentate gyrus and a loss of dendritic complexity in the mature neurons extending processes into the molecular layer and CA3 [29], [30]. While *Disc1* ^31L/31L^ mutants have proliferation deficits and a degree of dentate gyrus thinning (though not reaching statistical significance) consistent with *Npas3* null mice, only *Disc1* ^100P/100P^ mice have similar changes in neuronal morphology. We can conclude that both neural progenitor proliferation and immature neuron integrity influence dentate gyrus thickness.

### Cell proliferation and depression-related phenotypes

**Figure 5:**
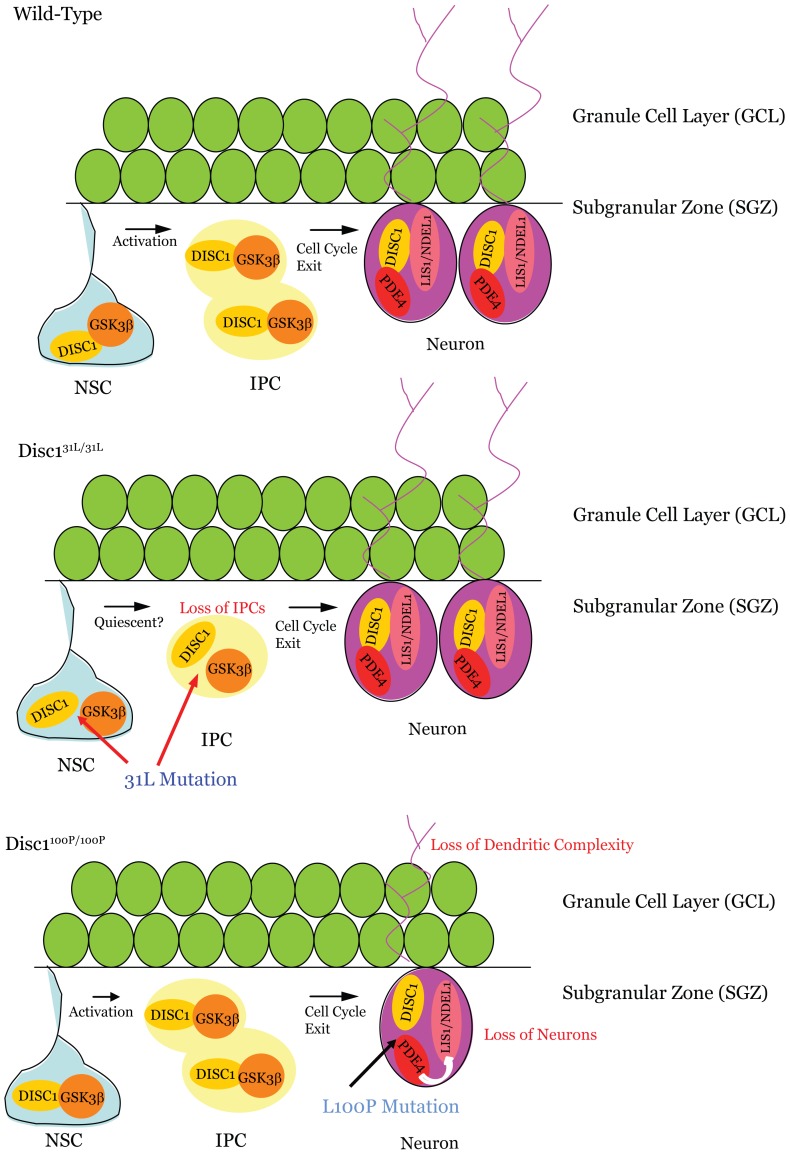
Hypothetical model for how DISC1 mutation may affect interacting proteins within the cell populations residing in the dentate gyrus. Neural stem cells (NSCs) generate neurons which extend processes across the granule cell layer through the generation of Tbr2^+^ intermediate progenitors (IPCs) as shown in the ‘wild type’ illustration. Our data suggests that the homozygous Q31L mutation in DISC1 leads to a loss of IPCs which may arise from a reduction in the formation of DISC1-GSK3β complexes in the NSCs leading to increased quiescence. With the homozygous L100P mutation, however, we note a normal number of IPCs, but instead observe alterations in the morphology and migration of the DCX^+^ postmitotic neurons, which we hypothesize may be due to changes in the NDEL1-LIS1 complex mediated by DISC1 interactions with PDE4.

Several independent groups have examined behavioral phenotypes in mice after selective changes in adult neurogenesis, but the results are inconclusive due to experimental differences and timings [31]–[34]. Chronic unpredictable stress, a paradigm for inducing depression-related phenotypes, which necessitates exposing rats or mice to multiple stressors for several weeks, leads to a loss of early neural stem cell (NSC) progenitors and more neuronally committed progenitors in the adult SGZ, supporting a link between depression and decreased neurogenesis [34]. Acute stressors acting on mice for only 45 minutes, however, only affect the proliferation of NSCs in the SGZ, and spare the more neuronally committed progenitors [34]. The inference is that the critical cell population affected through stress induced depression is the NSCs, and the losses of downstream progenitors are a secondary effect and a consequence of a long term loss in NSC proliferation [34]. When we examined NSC proliferation in the adult hippocampus using the neurosphere assay, we noted deficits only in the *Disc1* ^31L/31L^ mice, which have depression-related phenotypes. Neurospheres formed from adult hippocampal tissue require Hes5, a marker for NSCs that is not expressed in later proliferating cells and postmitotic neurons [17]. Thus, we can hypothesize that the loss of more neuronally committed Tbr2^+^ cells in the *Disc1* ^31L/31L^ mice is a secondary effect. The majority of NSCs in the adult hippocampus are quiescent, however, and not actively dividing and generating new neurons [16], [35]. Therefore, it seems possible that DISC1 is either altering the balance between the quiescent and active NSC population, or simply blocking NSC division into downstream proliferating cell types. The data from Mao et al (2009), which show that the DISC1-GSK3β complex regulates cell proliferation [4], provide a framework for understanding how the Q31L mutation is potentially mediating the loss of Tbr2^+^ intermediate progenitors (Figure 5) since it maps to the disordered N-terminus of DISC1 that contains the expected binding site to GSK3β [2], and has been shown to disrupt the DISC1-GSK3β complex [36].

### Newly generated neurons and schizophrenia-related phenotypes

*Disc1* ^100P/100P^ mice were more active in the open field, and had more severe deficits in working memory and prepulse inhibition (PPI) than the *Disc1* ^31L/31L^ mice [11]. The critical population of cells mediating many hippocampal-dependent memory tasks appears to be the newly born immature neurons in the SGZ [37]. In mice, reductions in the number of immature neurons or premature differentiation of progenitors lead to a loss in neuronal complexity and leads to mice displaying deficits in spatial memory tasks [31], [32]. The reduction in DCX^+^ cells and less developed dendritic arbors that we noted only in the *Disc1* ^100P/100P^ mice (Fig 3C) thus may correlate to the more severe deficits in working memory described previously [11]. This supports two separate studies on mice lacking the full length DISC1 protein that display working memory deficits without major changes in other tests of hippocampal learning [15], [38]. Notably, in mice carrying a targeted disruption of the endogenous *Disc1* allele, resulting in a truncated N-terminal protein, working memory deficits accompany morphological changes in the immature neuron population that are linked to elevated cAMP levels [15], [27].

Our observation of allele specific phenotypes arising from different *Disc1* mutations with otherwise identical expression levels throughout development provides an opportunity to distinguish possible molecular mechanisms. The contrasting effects of the DISC1 mutations we observe in the hippocampus differ from a study utilizing the same line of *Disc1* mice to examine how DISC1 affects the embryonic cortex. In that report, both DISC1 mutations led to similar changes in neuronal morphology, spine density, and distribution of neurons in the cortical layers [13], suggesting that proteins within the DISC1 interactome that are affected by these missense mutations are uniquely modulated within select cell populations. To date, more than 30 different proteins have been shown to have specific DISC1 interacting sites, suggesting that the DISC1 mutant proteins likely modulate multiple signalling pathways [39]. While it is unclear which of several potential N-terminal protein interactors may play a conjoint role with DISC1 with respect to the changes observed in the DCX^+^ neurons, it is notable that the L100P strongly disrupts binding of DISC1 to PDE4B [11], and the DISC1-PDE4 complex can regulate neurite outgrowth by modulating the LIS1/NDEL1 complex [3]. As both LIS1 and NDEL1 are critical for neuronal branching and migration [8], we can formulate a model by which the L100P mutation modulates PDE4B activity, which then suppresses neurite outgrowth through changes in the LIS1/NDEL1 complex (Figure 5). Our results, by illustrating how missense mutations in DISC1 can lead to specific cellular phenotypes in the hippocampus, support a role for DISC1 variation in brain development, capacity and mental disorder.

## Materials and Methods

### Mice

All animal experiments were approved by a University of Edinburgh internal ethics committee, and all procedures were performed in compliance with UK Home Office regulations (project license number 60/4179). Wild-type mice and the two *Disc1* homozygous mutants were generated as described previously [11]. Congenic strains of *Disc1* ^WT/WT^, *Disc1* ^100P/100P^, and *Disc1* ^31L/31L^ were generated by repeated (n = 12) backcrossing to the C57BL/6JRcc strain (Harlan, Bicester, UK). Mice were housed in a 12 hour light/dark cycle and fed a regular diet *ad libitum*.

### Antibodies

The following antibodies were used at the specified dilution: rabbit anti-doublecortin (DCX) (1∶400, Abcam), rabbit anti-Tbr2 (1∶200, Abcam), and rabbit-anti-activated caspase 3 (1∶100, Abcam). Disc1 was detected using an in-house generated rabbit anti-Disc1 antibody (SK6479, used at 1∶2000 dilution) raised to amino acids 666–852 of mouse Disc1. Full details of the generation and characterization of this antibody will be provided elsewhere (Ogawa et al, in preparation). Vinculin was detected using mouse anti-Vinculin (ab18058 Abcam, used at 1∶2000 dilution).

### Immunohistochemistry

For counting Tbr2 and DCX immunopositive cells, every sixth section taken from 20 µm thick coronal sections of the dentate gyrus was processed for immunohistochemistry. The entire region sectioned was approximately 2.5 mm, so that there were between 18–20 sections immunostained per mouse, which were then counted using a blinded and randomized system. Sections were incubated for 2 hours at room temperature in blocking buffer (BB) (PBS with 5% normal goat serum, 3% BSA and 0.2% Triton-X-100) before incubating overnight at 4°C in the primary antibody diluted in BB. Following three washes with PBS, sections were incubated with the appropriate Alexa conjugated secondary antibody (Invitrogen, CA) for 1 hour, counterstained with Hoechst 33342 and mounted onto slides with Fluoromount-G (Southern Biotech). Antigen retrieval before blocking was performed by immersing the slides in preheated 10 mM citrate buffer and boiling at low power in a microwave for 10 minutes. For cell counts, every sixth section from the dentate gyrus was processed.

### Analysis of Dendritic Complexity and Migration

The complexity of dendritic arbors of immature neurons immunopositive for doublecortin (DCX) was assessed using ImageJ (http://imagej.nih.gov/ij/) utilizing a Sholl analysis plugin (Ghosh Lab Website, http://biology.ucsd.edu/labs/ghosh/software/ShollAnalysis_.class) with 10 µm separating concentric circles that centred at the cell soma. ImageJ was used to threshold the confocal z-stack projections of DCX^+^ arbors prior to using the plugin. Only neurons with clearly identifiable processes extending across the granule cell layer were selected for Sholl analysis. Due to the overlap of dendritic arbors in neuron dense areas in the dentate gyrus, the number of intersections was divided by the number of cell bodies. A comparison was made between the results of this estimation versus traces of neuronal morphology made in Adobe Photoshop. We observed a slight underestimation of the number of dendritic intersections calculated per neuron by this method, but the same trend between genotypes. Positioning of DCX^+^ cells across the granule layer was assessed by converting the distance spanning the granule layer and molecular layer into 10 µm bins and assigning bin values to each migrating cell. DCX+ cells were only scored if they were found a minimum of 20 µm away from the centre of the subgranular zone. All images of DCX^+^ cells analyzed were z-stack projections of 0.5 µm slices taken at 40x magnification on a Leica confocal SP3.

### Hippocampal Measurements

Paraformaldehyde perfused brains were sectioned at 20 µm in the coronal plane, stained with Hoechst 33342 to label nuclei, and imaged on a Leica confocal SP3. Measurements of dentate gyrus thickness were made in the upper blade where the dentate gyrus is parallel to the brain surface. For each brain, measurements were taken at every sixth section spanning the dorsal to ventral boundaries of the dentate gyrus.

### Neurosphere Colony Forming Assay

Hippocampal tissues from adult three-month-old male mice (*WT* = 6, *Disc1* ^31L/31L^ = 6, *Disc1* ^100P/100P^ = 7) were dissected, separated from the meninges, triturated, and incubated in 1 ml of papain solution (1200 units Papain, 100 µg DNase I Type IV (Sigma), 600 µg L-cysteine) at 37°C for 1 hour. Digested tissue was neutralized with a basic high serum media (Dulbecco's modified Eagle's medium (DMEM) with high glucose, 2 mM L-Glutamine (Gibco), penicillin-streptomycin (Gibco), and 10% fetal calf serm). Cell pellets obtained after a 5 minute 1200 g spin were resuspended in neurosphere media (DMEM/F12 with pencillin:streptomycin, B-27 supplement (Gibco, Baltimore, MD) plus 10 ng/mL EGF and 10 ng/mL FGF). The number of neurospheres formed after 7 days was counted from cells diluted to a concentration of 20 cells/µl and placed in a 96 well plate. This dilution was chosen since over 80% of the wells in the 96 well plate at this concentration in the wild-type controls generated only a single neurosphere.

### Brain lysates

P90 female wild-type, 31L and 100P mice were culled by cervical dislocation and brains were collected in ice cold PBS. For each brain the hippocampus and cortex were dissected, snap frozen and stored at −80°C. Per group the hippocampi and cortices of three mice were pooled and lysed in PBS with 1% triton X-100 and freshly added protease (Roche Complete) plus phosphatase (Calbiochem Set II) inhibitors. Lysates were centrifuged at 13,000 rpm for 10 minutes at 4°C in a bench top centrifuge. P7 wild-type and Disc1 exon 2-3 knockout (Δ/Δ exon 2-3) mouse brain lysates generated as described previously [14], were gifted by Professor Kozo Kaibuchi (Nagoya University). Protein concentrations were measured using a Bradford protein assay (Biorad).

### Immunoblotting

Equal amounts of protein were loaded onto a 6% Bis-Tris polyacrylamide gel. Separated proteins were transferred onto polyvinylidene difluoride membrane membrane (GE Healthcare) using semi-dry transfer (Biorad) in Tris-Glycine-SDS buffer with 20% methanol. Membranes were blocked in TBS-T (50 mM Tris–HCl (pH 7.5), 150 mM NaCl and 0.1% Tween-20) containing 5% skimmed milk for 30 minutes at room temperature. Incubation with primary or secondary antibodies was carried out in PBS-T with 1% skimmed milk overnight at 4°C or for one hour at room temperature, respectively. Protein bands were detected by incubation with peroxidase-conjugated secondary antibodies (DAKO) for 45 min at room temperature and visualized using ECL or ECL-2 reagent (GE Healthcare). To assess protein loading following probing with the anti-Disc1 antibody, the membrane was stripped using Restore Western Blot stripping buffer (Thermo Scientific) for 30 minutes at room temperature, followed by washing in TBS-T.

### Statistics

To assess statistical differences between the wild-type and mutant *Disc1* lines, a one-way ANOVA was used with an appropriate *post hoc* test for multiple comparisons (when there was a p<0.05) to compare between genotypes. A Student's two-tailed unpaired t-test was used to confirm significance and compare differences between only two groups. For Sholl analysis, a two-way repeated-measures ANOVA was performed with genotype and distance as the two factors.
